# Exploring the role of nurses in after-hours telephone services in regional areas; A scoping review

**DOI:** 10.1371/journal.pone.0237306

**Published:** 2020-08-24

**Authors:** Adele Baldwin, Eileen Willis, Clare Harvey, Melanie Lang, Desley Hegney, David Heard, Brody Heritage, Jamin Claes, Denise Patterson, Venessa Curnow

**Affiliations:** 1 School of Nursing, Midwifery and Social Science, Central Queensland University, Townsville campus, Townsville, Queensland, Australia; 2 Research Division, Central Queensland University, Brisbane campus, Brisbane, Queensland, Australia; 3 School of Nursing, University of Adelaide, Adelaide, South Australia, Australia; 4 College of Science, Health, Engineering and Education, Murdoch University, Perth, Western Australia; 5 Cairns Hospital and Health Service, Cairns, North Queensland; 6 Torres and Cape Hospital and Health Service, Cairns, North Queensland; Technion - Israel Institute of Technology, ISRAEL

## Abstract

**Introduction:**

The management of patients who need chronic and complex care is a focus of attention internationally, brought about by an increase in chronic conditions, requiring significantly more care over longer periods of time. The increase in chronic conditions has placed pressure on health services, financially and physically, bringing about changes in the way care is delivered, with hospital avoidance and home-based care encouraged. In this environment, nurses play an important role in co-ordinating care across services. This review formed one part of a funded project that explored the nurse navigator role within a proposed 24-hour telephone-call service in one regional area that has a diverse population in terms of cultural identity and geographical location in relation to service access.

**Aim:**

The review reports on the extant literature on the nurse’s role in the provision of afterhours telephone services for patients with chronic and complex conditions. The specific aim was to explore the effectiveness of services for patients in geographically isolated locations.

**Methods:**

The methodological approach to the review followed the Preferred Reporting System for Meta-Analyses (PRISMA) guidelines. A thematic analysis was used to identify themes with chronic care models underpinning analysis.

**Results:**

Three themes were identified; nurse-led decision making; consumer profile; and program outcomes. Each theme was divided into two sub-themes. The two sub-themes for decision making were: the experience of the staff who provided the service and the tool or protocol used. The two sub-themes for consumers profile were; the geographic/demographic identity of the consumers, and consumer satisfaction. The final theme of outcomes describes how the effectiveness of the service is measured, broken into two sub-themes: the economic/workforce outcomes and the consumer outcomes.

**Discussion:**

The provision of an after-hours telephone service, in whatever model used should align with a Chronic Care Model. In this way, after-hours telephone services provided by experienced nurses, supported by ongoing professional development and relevant protocols, form part of the ongoing improvement for chronic and complex care management as a health priority.

## Introduction

Chronic disease is commonly defined as any long-term condition that imposes significant functional limitations upon the individual, which worsen over time to negatively affect their quality of life [1]. Experts caution that the term ‘chronic condition’ should be used rather than the term ‘chronic disease’ as it refers to more than just the disease process, and because it includes reference to associated injuries and disabilities [2] requiring a well thought out, structured approach from all levels of health providers [3]. One strategy, to assist people with chronic conditions to avoid preventable hospital admissions, has been the establishment of the Nurse Navigator role. Nurse Navigators are experienced registered nurses, with specialised skills in a health area, employed to guide patients across health services and departments, thus helping them to navigate complex health systems. This role fits within the changing focus of the Chronic Care Model and the Innovative Care for Chronic Conditions Framework [3] p. 4.

The project that this review underpins was funded by a collaborative grant from a regional primary healthcare network (Australia) and aligns with a larger study that evaluates the role of the Nurse Navigators in the region. The role description and work of Nurse Navigators varies across health services, however, in the region where this project is placed, they also provide an out-of-hours telephone advice service until 10pm, seven days a week. Hence, this review is the initial step in the project aimed at establishing an afterhours mobile (cell) phone healthcare service in a rural/remote area of Australia. The review is registered in PROSPERO [4]. The methodological approach to the review followed the Preferred Reporting System for Meta-Analyses (PRISMA) guidelines and all papers selected for final analysis were assessed for rigour, credibility, and validity using the Critical Appraisal Skills Programme (CASP) tools relevant to the research design within the publication [5, 6].

Recent Australian data estimates that around 73% of all deaths are attributable to chronic conditions [7]. The incidence of chronic and complex conditions is increasing globally with Australian statistics estimating more than 11 million people [1], or up to 50% of all Australians are diagnosed with at least one chronic condition. Further, almost one in three people over the age of 65 years report having a diagnosis of a chronic condition [1].

At the same time, there is increasing pressure on health budgets which already account for a large percentage of GDP for all countries [1, 8]. Primary and preventative health strategies are highlighted areas to reduce the strain on limited government resources and the financial pressure on individual families, particularly in low income countries [9]. Calculating the financial cost or burden of chronic conditions is complex and the factors included in the analysis are not exhaustive, but often include so-called unnecessary admissions to hospital [10]. It is estimated that around 39% of preventable hospital admissions are from patients with at least one chronic condition. Thus, it is generally accepted that if disease incidence can be improved by changing health behaviours, and chronic conditions can be managed outside institutions, the cost benefit is substantial [11].

Accordingly, health services are setting targets for hospital avoidance and shifting to home-based care, strategies that are imperative given the previously outlined escalating costs of care, increasing chronicity as well as an ageing demographic [12, 13]. Factors associated with re-admissions are largely due to poor health literacy, low socioeconomic status (including homelessness), geographic isolation and cultural differences. The inequalities associated with chronic conditions are apparent across socioeconomic groups and geographic location [1] with people from lower socioeconomic groups and those who reside in rural/remote areas more likely to have higher rates of chronic and complex conditions. A recent report stated that the burden of disease would be less if people from rural/remote areas were afforded the same access and opportunities to health services as those who live in metropolitan/regional areas [7].

A significant number of patients in rural/remote communities in Australia, particularly in Northern Australia, identify as Aboriginal and/or Torres Strait Islander peoples, and have an added health disadvantage and are over-represented in the chronic condition data [14]. One strategy within the complex and multi-level national Australian Closing the Gap strategy [15] for meeting the social and health needs of Aboriginal people is the use of outreach-type systems, using telephone technology after-hours to better support people who cannot physically access care facilities for whatever reason. However, some reported research states that despite the growth in primary health care provision, the utilisation by low-socio economic populations is not comparative, identifying that the social determinants of health have a major impact on access to health care [16].

### Aim

Given the issues highlighted above, this scoping review aimed to better understand existing models and current evidence of such services. A scoping review was selected as research related to nurse navigators providing after-hours telephone services remains unclear with terminology imprecise, and the area relatively new [17]. The review was conducted between March and August 2019, and addressed the following questions:

- What models of care are used to provide after-hours services to patients with chronic and complex conditions?
- Are there models of nurse-led after-hours care and are they effective?
- Are there any models or systems of after-hours care for patients with chronic and complex conditions that address geographical isolation and are they effective?

## Method

Although a scoping review does not require an ethical clearance, this project within which this review was undertaken has been ethically approved by Queensland Health HREC/18/QTDD/8 and by Central Queensland University Human Research Ethics Committee Ethics No. 020740 "The evaluation of the nurse navigator/ health navigator: a trans-Tasman study".

The study followed the protocols for scoping reviews as outlined by Munn et al. [17]. The population for the inquiry was nurses in co-ordinator type roles, who provide after-hours telephone services to patients with chronic and complex conditions. The search terms used were after hour* OR 24 hour* AND triage AND rural OR remote OR isolated OR regional and were applied to the academic databases of CINAHL, Proquest, Medline, Scopus, Embase, and Web of Science. The same search was applied to the grey literature. The initial searches of all sources included the term ‘nurs*’. However, this added refinement significantly limited the results, thus, the review team amended the search terms to those above without the limitation of ‘nurs*’.

**Fig 1:**
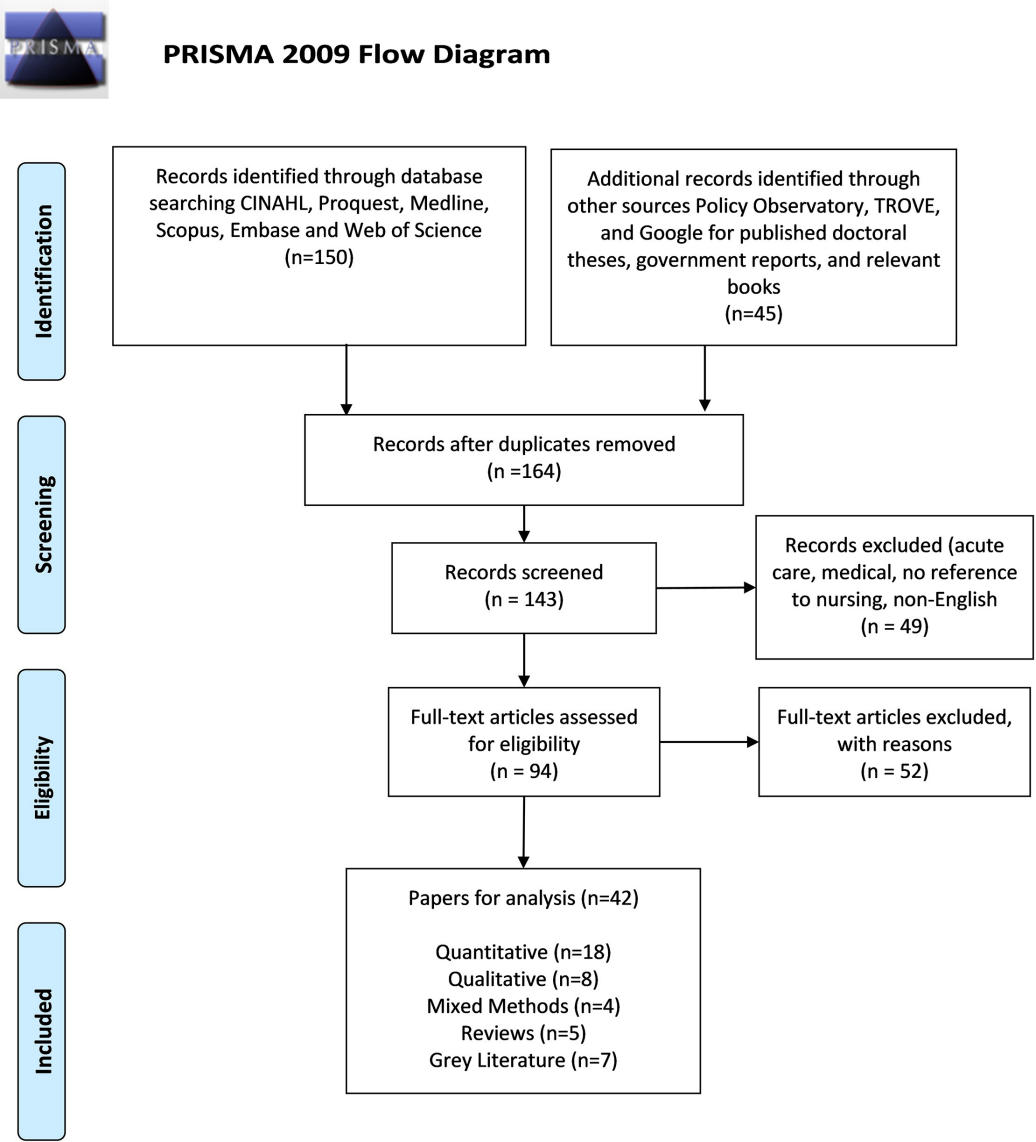
PRISMA flowchart.

Additionally, minor modifications to the search terms were made to fit the search engines for the grey literature which was limited to four data bases; Proquest (Dissertations and Theses), the Policy Observatory, TROVE and Google. These data bases were selected because they provide access to recently published doctoral theses, government reports, and relevant books. The inclusion and exclusion criteria for this review, as per PROSPERO (record ID 105594), were English peer reviewed and grey literature (government, non-government organisations) published between 2000–2018, that dealt with chronic and complex care, in regional, rural and remote areas. Papers published before 2000, that dealt with acute care settings, that did not relate to medical or nursing issues and were not in English were excluded. Inclusion of the grey literature extended the scope of this review, recognising that relevant and important knowledge exists outside of academic databases. Accepted papers were organised according to the three-level hierarchy with only Tier One (books, government reports, thesis, and white papers) accepted [18]. The search is represented in the PRISMA flowchart (Fig 1).

In total, the full text of 145 papers were downloaded and screened, of which 95 were assessed for eligibility within the search criteria. Of these, 42 were subject to further analysis, as shown in Fig 1 the PRISMA flowchart.

The final 42 papers were categorised according to the hierarchy of evidence moving from the five systematic literature reviews to cross sectional studies, qualitative research and grey literature. As shown in the data summary table, this included four literature reviews [19–22], and one Cochrane review [23]. The quantitative studies ranged from randomised controlled trials and interventions [24, 25] to, pre and post-test interventions [26–28] to cross sectional and longitudinal studies [29–36] to studies that employed surveys and questionnaires [37, 38] and analysis of case records [39, 40]. There were three mixed method studies [41–43] that drew on existing call centre data and interviews, as well as six qualitative studies. The qualitative studies were interviews with consumer/callers or call takers [44–51]. The final seven papers from the grey literature, included one book chapter, four PhD theses, one Master of Science thesis, and one report [52–58]. These papers follow the hierarchy of evidence as outlined by Daly and colleagues [59]. The final papers are included in Table 1, the data summary table, below.

**Table 1:** Data summary table.

Authors, year, location	Population	Design methods	Findings/Comments
**LITERATURE REVIEWS**			
1.Bunn, F., Byrne, G. & Kendall, S. (2004). UK	Cochrane review	Review that included RCTs, controlled studies, controlled before/after studies, interrupted time series.	Most of the telephone consultations are done by nurses using a computer-based clinical decision support system.
			Focussed on NHS Direct- a 24 hour nurse-led system
			Underuse of NHS Direct by older people, ethnic minorities and other disadvantaged groups
			Decrease in demand for immediate GP review or home visits
			50% of calls can be handled by telephone advice alone
		Disease specific phone lines were excluded	Telephone consultation and triage systems were terms used interchangeably
			Nurses can decrease the GP workload without an increase in adverse events
2.Devi, B., Syed-Abdul, S., Kumar, A., Iqbal, U., Nguyen, P., Li, Y. & Jian, W. (2015). Taiwan	Papers about mHealth published from 2005 onwards.	Literature search in academic databases restricted to HIV/AIDS and tuberculosis	Very few studies focused on key populations
			6 main categories: medication adherence; quality of care; prevention; education; data collection; disease monitoring
	90 articles		
			Most studies reported positive effects on medication adherence; treatment; promote education(sic); continuum of care
			Can decrease disease burden on healthcare system with more efficient prevention; treatment; education; data collection; management support
3.Huibers, L., Smits, M., Renaud, V., Giesen, P., & Wensing, M. (2011). No location for this study.	13 observational studies	Systematic Review”Studies were included if they concerned out-of-hours medical care and focused on telephone triage in patients with a first request for help”	Examined safety of advice provided via telephone triage service, with approximately 10% of standard calls being provided with unsafe advice, and 50% of simulated high-risk patients being provided with high-risk advice (argued that former is more valid though). Note importance of being able to identify high-risk patients more accurately/quickly via need for different training practices. Noted most literature on this subject quite old at time of review (2010).
4.Ismail, Gibbons & Gnani, 2013 UK	34 articles were reviewed	Systematic Review	The aim of the systematic review was to review the evidence on primary care service interventions to reduce inappropriate A&E attendances. Studies showed a negligible effect on A&E attendance for all interventions; data on patient outcomes and cost-effectiveness are limited.
5. Mbuagbaw, L., Mursleen, S., Lytvyn, L., Smieja, M., Dolovich, L. & Thabane, L. (2015) Global review	9 systematic reviews reporting on 37 source studies in 19 different countries	Overview of systematic reviews	Evidence supports the use of text messaging as a tool to improve adherence to medication and attendance at scheduled appointments.
			Mobile phone technology is emerging as a tool for chronic disease management
			Current evidence supports 2 way text messaging
			These authors raise the issue of confidentiality
**QUANTITATIVE STUDIES**			
6. Asiki et al. 2018 Uganda	Uganda pregnant women	Non RCT Intervention	The main objective of the intervention was to link pregnant women to their nearest primary health care facility during childbirth using smart phones to reduce home births and associated complications. In the adjusted analysis, the intervention associated with lower odds of home births [AOR = 0.38, 95%CI (0.15±0.97)].
		Logistic regression analysis	
7. Mangwi Ayiasi et al. 2016 Uganda	A total of 1385 pregnant women were analysed: 758 and 627 in the control and intervention respectively.	Demographics, structural questionnaires were used to collect data on maternal & newborn practices	The objective of this study was to measure the effect of home visits combined with mobile phone consultation on maternal and newborn care practices.
			Significant post-intervention differences were: delivery place [adjusted Odds Ratio aOR: 17.94(95%CI: 6.26–51.37); p<0.001], cord care [aOR: 3.05(95%CI: 1.81–5.12); p<0.001] thermal care [aOR: 7.58(95%CI: 2.52–22.82); p<0.001], and timely care seeking for newborn illness [aOR: 4.93(95%CI: 1.59–15.31); p = 0.006].
8. Bolli, S., Van Melle, G. & Laubscher, B. (2005). Switzerland	Nationwide postal survey of 38 Swiss public paediatric hospitals	Analysis of call records	Most units ran a nurse-led telephone triage advice system
			Only 50% of responding organisations gave specific training to staff
			Time distribution analysis–greater numbers of calls during winter, weekdays and early evenings and midmorning weekends and public holidays
9. Derkx, H. P., Rethans, J. J. E., Muijtjens, A. M., Maiburg, B. H., Winkens, R., Van Rooij, H. G., & Knottnerus, J. A. (2008). Netherlands.	17 after-hours call centres; researchers presented 9 standardised patient profiles, 3 times to each centre during a year.	Cross-sectional, descriptive analysis of call logs.	Found lower than anticipated frequency of critical questions asked by telephone nurse when calls were made; decisions were being made before sufficient information was gathered in many cases. Safety advice / home management advice was less frequently provided, or was less often correct.
10. Hildebrandt, D. E., Westfall, J. M., Fernald, D. H., & Pace, W. D. (2006). USA.	4949 call records, 2835 of these were after-hours clinical.	Cross-sectional, records from dataset of one practice.	Approximately a quarter of patients who were unable to reach their physician via the after-hours service continued to experience pain/discomfort, although the rate of follow-up (visiting a clinic following the call) was generally high. Interesting to note that the call service asks clients whether they are experiencing an ‘emergency’ (patient decides), and only then the call gets forwarded to a physician immediately; consequence of answering script and who ends up doing the triage over the phone.
11. Jeong, S., Choi, H., Gwon, S., Kim, J. South Korea	Low income older adults with hypertension and type 2 diabetes mellitus (n = 15 control group; n = 20 intervention group)	Randomised, controlled, single-blinded, parallel pilot trial Implied ethics approval	Telephone support for older adults is inexpensive and comfortable
			Telephone support is effective for behavioural modification for hypertension, diabetes, metabolic syndrome, post-operative symptoms
			Telephone interventions help to integrate information and increase self-care behaviours Terms: tele home care/telemonitoring
			Engagement of older adults was influenced by health literacy and digital literacy
			Telephone support by nurses should be regular, planned, structured service
			Trans theoretical model
12. John, M., Samson-Akpan, P., Akpabio, I. & John, E. (2016). Nigeria	Non-disclosed youth with HIV (n = 19)	One group Pre test/post test experimental design	Applied the Transtheoretical Model for self-care
			This population preferred mobile phone for counselling as more convenient, faceless, privacy and puts them at ease
13. Lee et al. 2002 Indonesia	Children requiring after hours health advice	Randomized interventional phone service with survey	Callers were less satisfied with medical advice provided by a nurse advice service compared with the traditional on-call paediatrician. The lower satisfaction was associated with somewhat poorer compliance with recommended triage dispositions and more frequent repeat calls for medical advice
	560 callers were enrolled in the on-call paediatrician group, and 616 were enrolled in the advice nurse group.		
14.McKenzie, R., Williamson, M., & Roberts, R. (2016). Australia.	Approximately 300000 calls.	Historical records from 2011–2013 of Healthdirect telephone service in Australia.	Lower socioeconomic advantage was associated with higher use of the service, while older adults and people in more rural areas were less frequently using the service. Parents, Aboriginal and Torres Strait Islander people, people in remote areas (note contrast with rural people prior) and women made more frequent use of the service. Suggest promotion of the service to people using it less-frequently may assist with better healthcare.
15. Navratil-Strawn, J., Ozminkowski, R. & Hartley, S. (2014). USA	Triage calls to a specific service in 2012 (n = 132 509)	Multiple methods of statistical analysis applied	57% of callers were compliant with nurse recommendations
			Average cost of $328 lower among compliant than non-compliant
			Telephone triage services should be staffed with experienced, well trained nurses with good communication skills; structured protocols; clinical algorithms
			Reasons for not complying with recommendations to attend ED include the cost, transport issues and change in the patients’ physical condition
			Caller compliance with nurse triage recommendations resulted in lower health care expenditures
16. Nonaka, D., Pongvongsa, T., Nishimoto, F., Nansounthavong, P., Hongwei, J., Vongsouvanh, A., … & Kobayashi, J. (2014). Laos, Central Asia.	154 village health workers / 11 village health worker supervisors.	Longitudinal, pre/post/follow-up design.	Distribution of mobile phones to remote village health workers improved the frequency in which monthly reports from villages were being sent through to central supervisors, and this improvement was evident in the follow-up measurement period. Advice seeking (e.g., treatment advice, service delivery) was a secondary facet of the calls that increased.
17. Ong, R. S., Post, J., Van Rooij, H., & De Haan, J. (2008). Netherlands.	6764 calls to two phone services; one using an expert system, one not.	Cross-sectional; archival/historical data from call records.	Found longer call duration for hub using expert system, although noted reduction in GP referrals (via phone or visit) and more calls sorted out by the assistant taking the call. However, they noted that GPs were liaised with during the call in the expert system, which may have contributed to the lack of requested GP follow-up.
18. Quan, V., Hulth, A., Kok, G., & Blumberg, L. (2014). South Africa.	1 nurse; 3–41 other villages across different categories (clinics with mobile reporting already in place; those without mobile reporting with same nurse; those without mobile reporting and different nurse)	Mixed design, but all quant; compared nurse reporting of malaria cases with/without mobile reporting, and compared mobile/non-mobile reporting frequency across other villages.	Generally found faster reporting speed / follow-up speed in instances where mobile reporting of malaria cases was undertaken.
19. Raknes, G., Hansen, E. H., & Hunskaar, S. (2013). Norway.	141 342 call records.	Cross-sectional, historical/archival records from after-hours phone service.	Found evidence of relationship between increasing geographic distance and reduced likelihood of using the telephone triage service (i.e., 50% reduction when patient is 43+ km away from metropolitan centre). Participants were more likely to be older, less likely to have acute cases, and less likely to be female as geographic distance increased from a metropolitan centre.
20. Reid, J., & Porter, S. (2011). UK.	7498 calls made to chemotherapy helpline	Cross-sectional, historical/archival data from phone logs.	Most calls were made by patients, by female patients, and during business hours. Advice provision was the most frequent course of action in response to a call. While patients were most common callers, a variety of professional/carer calls were also received, indicating service had broad uptake.
21. Stacey, D., Green, E., Ballantyne, B., Skrutkowski, M., Whynot, A., Tardif, L., … & Carley, M. (2016). Canada.	105 surveys regarding impression of the call service from patients/family.	Cross-sectional, survey data from callers of service.	Short Questionnaire for Out-of-hours Care (SQOC) might be used to assess caller impressions of the quality of service received from an after-hours call service. Found patients were generally OK with how calls were handled, although note in this study only an answering service / return of call following day was provided for after-hours callers.
22. Tran, D. T., Gibson, A., Randall, D., Havard, A., Byrne, M., Robinson, M., … & Jorm, L. R. (2017). Australia	8406 adults >45 years of age per a health helpline.	Cross-sectional, historical records from linkage dataset	Variations in patient compliance with advice provided by telephone triage service depended on patient characteristics (e.g., age) and psychological/lifestyle factors (e.g., anxiety, remoteness). While advice was provided via the service, the behavioural follow-up of visiting ED / seeing a doctor / self-care varied notably.
23. Watson, A. & Poima, R. (2015). Papua New Guinea	A total of 113 health-related phone calls were received during the first nine weeks of the call centre’s operation	Descriptive statistics	A total of 113 health-related phone calls were received during the first nine weeks of the call centre’s opera-tion. Most of these calls were from the public, while a small number were from rural health workers. Prank calls and calls enquiring about the service were also received. During establishment, mental health was not considered and calls that may fall into this category have not been logged separately.
**MIXED METHODS STUDIES**			
24. Croese et al. 2018 Australia	220 patients Post op patients in outer Cairns region	Study Survey **(no ethics, patients not aware of the study)**	38 (17%) completed the study survey. The majority of responses were positive in favour for the use of the nurse-led phone clinic. 95% of patients felt that a phone call is acceptable, 89% were satisfied with their follow-up and 81% felt that this form of follow-up should be considered by other hospitals. A total of 13% of respondents would have preferred to be followed up by a doctor.
25. De Coster, C., Quan, H., Elford, R., Li, B., Mazzei, L. & Zimmer, S. (2010). Canada	Analysis of existing Nurse telephone advice line data (96 368 symptom based calls)	Analysis of existing call records for defined health services	3 types of service–symptom triage, health information, help with accessing services Nurses select one of seven recommendations:
			Priority–immediate medical attention Emergent–see health care provider within 0–4 hours Urgent–see health care provider within 24 hours 72 hour referral–see healthcare provider within 72 hours if symptoms persist Interim self care Health information No recommendation Based on callers’ description of symptoms, nurses choose a protocol and use it plus clinical judgment to select a recommendation ED follow through is higher for rural and lower in urban areas Patients with a cardiac complaint were more likely to follow advice to go to ED. Follow through is higher when patients perceived the nurse as helpful and collaborative and the caller understood the advice
26. Elliott, A., McAteer, A., Heaney, D., Ritchie, L. & Hannaford, P. (2015). Scotland	NHS 24 data	Quantitative and qualitative analysis of the existing caller data to NHS 24.	Commonest outcomes of calls was advice to visit an out-of-hours centre Demographic difference
27. Phillips, J. L., Davidson, P. M., Newton, P. J., & DiGiacomo, M. (2008). Australia.	Palliative patients; 55 calls. Qual participants were unknown in *N* outside of 8 carers.	Cross-sectional archival data of calls to palliative service. Interviews analyses via thematic content analysis.	Provides flowchart of how their telephone AH service works, which may be useful. Most calls had cases solved over the phone, with only a tiny amount recommending an ED visit. Most calls between 6pm-12am. Interview follow-ups found support for the service not just from patients/carers, but also from nurses/managers regarding its efficacy.
**QUALITATIVE STUDIES**			
28. Mangwi Ayiasi et al. 2015 Uganda	Uganda pre & post-natal women	67 interviews audiotaped and transcribed into English	This study aimed at exploring perceived maternal and newborn benefits of VHTs making home visits to prenatal and postnatal women and using mobile phone consultations to link VHTs to professional health care workers for further advice. Majority of women and VHTs contend that the intervention improved access to maternal and newborn information; reduced costs of accessing care and facilitated referral.
29.Egbunike, J. N., Shaw, C., Porter, A., Button, L. A., Kinnersley, P., Hood, K., … & Edwards, A. (2010). UK	60 services users	Purposive sampling (service users and carers); interviews + thematic analysis	When commenting on the efficacy of after-hours care, noted that compared to GP home-visits etc. telephone triage was less-preferable. In terms of service user satisfaction, factors such as timeliness of returning a call to a GP from an answering service, the quality of communication between caller and responder, and the amount of hurdles/steps taken to get advice via the service (more steps = worse) were notable.
30. Elsom, S., Sands, N., Roper, C., Hoppner, C., & Gerdtz, M. (2013). Australia	During the 3‐month sample period, July to September 2009, 128 calls were received from carers and 76 were received from consumers. Triage referral of mental health patients	Interviews	The main findings of the study were that consumers experienced more difficulty than carers in accessing the service and that, although most participants were satisfied, only a minority reported being involved in decision-making. Further work is needed to improve accessibility of mental health triage services and to investigate barriers to consumer self-referral. Professional development and practice support systems should be established to support mental health triage nurses in the development of collaborative, consumer focused care.
31.Hackett, K. M., Kazemi, M., & Sellen, D. W. (2018). Tanzania	This study was carried out within the context of a community-based maternal and child health project implemented by World Vision (WV), a large international non profit organization	Interviews	Researchers examined the perspectives of frontline community health workers and their female clients regarding data security and privacy within the context of an mHealth intervention to improve women's uptake of maternal health services from October 2013 to July 2014 in rural Tanzania. Qualitative findings demonstrate that the use of new technologies to capture health service user data during pregnancy and childbirth has both positive and negative impacts on perceptions of personal privacy and confidentiality.
32.Karari, C., Tittle, R., Penner, J., Kulzer, J., Bukusi, E., Marima, R. & Cohen, C. (2011) Kenya	296 calls to a hotline Documents related to HIV hotline	Phone consultant and healthcare workers	Heavily burdened areas are also the most under-resourced
			Differentiate for “consultation systems” Term “warm line” 2 barriers to use: Poor mobile phone coverage Slow response time of consultants
33.Knight, K., Kenny, A. & Endacott, R. (2015) Australia	RNs from five rural health services (n = 15)	Multicase study design using a qualitative descriptive approach	People have 24/24 access to formal telephone triage services but rural people continue to directly telephone local health services seeking care.
			Telephone as a connection to the community
			End the phone call often signalled the beginning of an episode of ongoing care; ending the call but not the encounter
			Rural nurses used extant knowledge of the callers, community and other healthcare providers.
			Nurses working in call centres do not have a pre-existing relationship
34. Lee, S., Chib, A. & Kim, J. (2011) Indonesia	223 midwives	Tested a theoretical model	Core role of information and communication technologies in developing communities enhanced communication and connectivity
	Midwives as community health care workers		
			Cell phones effective in rural and remote environments by providing benefits such as low-cost, deployment, flexible infrastructure and community-shared resources.
			Knowledge is transmitted through social networks to a larger extent in rural communities
			CHWs in developing countries create linkages between higher level health resources and patients
			Cell phones could be used as tools for better mobilising resources via enhancing communication structure within the community
			Effectiveness is dependent on the ability of the individual to access available resources
35. Roberts, A., Heaney, D., Haddow, G. & O'Donnell, C. (2009). UK	35 (NHS 24 stakeholders / partners of NHS boards)	Face-to-face / telephone interviews; constant comparative approach to analysis (grounded theory type?)	Noted nurse-led triage via phones did not fare well for the rural and remote service users. The national rollout of the NHS telephone triage service didn’t mesh with the rural/remote client expectations, and didn’t seem to take into account local variations in service delivery that were available. Gap between previous service offerings (doctor on call) and standardised national rollout of telephone service in rural/remote Scotland.
**Grey literature**			
36. Cariello, F. 2000	Pilot study of 30 managed care plan callers then 300 randomly selected care plan callers.	PhD study	Rate telehealth service nurses as responsive and empathetic.
		Telephone survey design to evaluate RN protocol for service using SERVQUAL tool	Reliability rated lower than responsive and empathetic
			Male patients give lower satisfaction
			Education of patient does not impact on satisfaction
			Overall satisfaction rate on scale of 7 is 6.5
37. Enomoto 2006	8 RNs interviews	Master of Science thesis development of algorithm and qualitative testing of it with RNs	The experienced nurse builds up a mental model of patient and their symptoms during telehealth
			More experienced nurses ask more questions on the phone during a telehealth consultation
			Experienced nurses get patients to do simple physical tests to make a judgement on patient responses; e.g. do they have pain when they bend forward
			The recommendations a nurse gives to a patient must match the resources available to the patient
			Is telehealth the nurses primary work, or are they doing it alongside other clinical activities Algorithm or decision support tool provides guided questions that assist in conclusion and recommendation to patient
			The design of any algorithm must have a boundary established that is based on the patients characteristics, for example, information on where the patient lives, will impact on decision to proceed to an emergency department or not given possible distances or access- or resources
			Protocols have three parts; a checklist to aid assessment; a decision algorithm to aid in recommendations and a symptom map to aid diagnosis
			Algorithms that provide a visual clue while nurse is gathering patient information helpful, but not seen to impact on recommendations or outcomes
38.Fonesca 2017	1) 368 oncology nurses	PhD thesis involving 1) Initial survey, 2) testing protocol, 3) small scale testing of protocol, 4) expanded testing of protocol and evaluation	The checklist algorithm needs to be efficient and simple to follow
	2) 5 Oncology Nurses		There is a danger that nurses will use the protocol/algorithm/check list too rigidly
	3) 5 Oncology nurses dealing with several patients over 5 days		
	4) 5 additional RNs over several days with number of patients over 5 days		
39. Greenberg 2005	10 RNS	PhD thesis. Grounded theory qualitative interviews	Protocols do not substitute for nurse experience
			Protocols often do not deal with patient complexity requiring the nurse to prioritise and deal with this complexity
			Protocols do not capture nursing knowledge about what to observe/ask or explore
40. Leaberry 2011	33 patients	PhD Prospective, longitudinal design	Calls were made by Heart Failure nurse practitioners
			The length of the questionnaire was a barrier as the patients tire quickly
			Improved quality of life, decrease in anxiety and depression, 46% reduction in readmission rates
			Significant reduction in hospital admission with remote telemonitoring
41. Offenbeek and Boonstra 2010	335 patients in video consults and 288 in home visits.	Journal article report of major study. The study was multi-method analysis of video consultations. Video consultations compared with home visits with patients over a 8 month period.	Nurses involved in the study were experienced RNs
			Telehealthcare does not substitute for a nurse visit
			Video consultations by the nurse with clients who volunteered for the service. No protocol was mentioned
			Patients satisfaction centred on the ‘safety net’ aspect of the telehealthcare program. They still wanted the nurse to visit
42. Vecellio 2013	Variable population depending on literature included in SLR.	Systematic literature review of quantitative and qualitative studies of nurse-led telephone triage	The evidence is inconclusive as to whether visual clues improve Telehealth patient assessment or recommendations, but may increase clinician satisfaction

The search team was divided between peer review and grey literature with the final papers selected by each team with one reviewer re-reading all papers. Then, the full review team identified the major themes that emerged from the selected literature. Several team discussions were held over a period of a month using the video conferencing tool Zoom, to identify themes allowing for common threads to emerge that were, in turn, grouped into sub-themes that addressed the review questions [60]. The grouping into themes and sub-themes was done by the first author and confirmed as part of the Zoom discussion.

### Findings

One of the major difficulties in the analysis of the findings, was the variety of terms used to describe the targeted service, making comparisons and analysis difficult. Terms included *mobile phone healthcare service*, *telephone consultations*, *mobile phone technology*, *nurse-led telephone triage*, *telephone support*, *telehomecare*, *telemonitoring*, *out of hours care*, *mhealth*, and *call centre care*. These terms are not mutually exclusive as in many instances they refer to similar services, while at other times they refer to different provisions. Programs in developing countries may use the mobile service to maintain contact with pregnant women between visits to reduce the number of home births or because the local health worker needs more expert support or is required to make regular reports best done via mobile services rather than mail [38]. Within developed economies these services may be established to reduce unnecessary visits to an emergency department or the patient’s primary physician [36], or act as a follow up service to patients discharged following surgery [55], and they may or may not operate after-hours. The skills required for nurses and other health workers for these various programs vary and were difficult to dissect given the variety of terms and approaches used.

Despite this variety of definitions, three distinct themes were apparent in the literature. These were: i) nurse-led decision making; ii) consumers profile; and iii) program outcomes. Each theme had two sub-themes. The two sub-themes for decision making were: the experience of the staff who provided the service and the tool or protocol used. The two sub-themes for the theme consumers profile were; the geographic/ demographic identity of the consumers and consumer satisfaction, and for the third theme, program outcomes, sub-themes were effectiveness and economic/workforce outcomes and consumer outcomes.

#### Theme 1 –Nurse-led decision making

Two of the key factors integral to the success of nurse-led telephone services are apparent in the reviewed literature and are key elements of the theme of decision making. Firstly, registered nurses need to be experienced, and secondly there is a consensus that they need tailored training in providing advice.

*Experience of staff*. Some researchers report that independently of support protocols (often a requirement to ensure compliance with registration) [53], nurses must also employ a high level of clinical judgement [35–37, 52, 54] and avoid slavishly using algorithms [53]. These findings suggest that telephone triage is a nurse speciality requiring education and access to expert advice. In spite of the fact that expertise in responding to calls was identified as necessity, Bolli et al. [40] report that only 50% of the telephone triage services offered staff training, and Huibers et al. [20] claim up to 50% of consumers receive incorrect advice. Huibers et al. [20] also point out that nurses providing telephone triage services require different training practices to develop the requisite skills to make quick, accurate clinical decisions. Knight et al. [51] warns of the need for clinical urgency when making an assessment over the telephone. Further, the nurses in the study reported by Knight et al. noted inconsistent compliance with policies and admitted using protocols selectively.

Although nurses may provide expert advice, in developing countries, for example, services were used as a support tool for village health workers in their day to day care of patients and it was the health worker who was in contact with distant experts [26, 27, 34, 38]. Within developed countries telephone services providing one-off health care advice to patients are usually staffed by experienced registered nurses [23, 52], although some are staffed by doctors, or provide a direct referral to a medical specialist [23, 33, 37, 40, 45].

The essential skills of nurses or health workers as call takers extend beyond their clinical assessment skills. Knight et al. [61] found that there is more to accurate assessment than clinical assessment and interpretation. These authors found that among the essential skills that nurses use to gather and interpret information, “listening and the art of conversation” are key. Additionally, Knight et al. proposed that nurses working in rural areas use their existing knowledge of the individuals and community as part of their decision making, highlighting the difference between locally driven triage services and central call centres. There are conflicting reports about the quality of the decision making in the literature. For example, Enomoto [52] found that the more experienced nurses asked more questions to elicit important information from the caller.

*Tools/protocols used*. Advice given is commonly based on computer-based support algorithms or protocols that provide a step-by-step set of questions that assist the nurse in assessing the patient and providing advice [23, 36, 37, 52–54]. Supporting the need for a protocol, Derkx et al. [35] found that some nurses made decisions about the patient’s condition before all the relevant clinical information was gathered, highlighting the need for appropriately prepared nurses and reinforcing that both the use of protocols and the clinical experience of the services provider (usually a registered nurse) are essential elements for phone-based triage/support. The research reported by Jeong et al. [25] includes a description of the development of an algorithm developed for the specific needs of their target population.

One of the unique points made by Karari et al. [50] was the idea of a ‘warm line’, a concept developed to meet the needs of a specific population in an under-resourced area. Warm lines are run by peers or volunteers and do not require the caller to provide their personal details. These authors also found that the barriers to use included poor mobile telephone coverage and slow response time by consultants; elements that are reflective of how wide ranging the term ‘under-resourced’ is. Despite lack of resources being a fact of life for many residents living in in rural and remote areas, regardless of the affluence of the country, sometimes is not given adequate weight in service planning. Lee et al. [24] provide further support for the development of suitable technological infrastructure with support from findings from their research into midwives as community care health workers. They state that mobile/cell phones are effective in rural and remote communities due to the low cost and relatively accessibility and may also be useful tools to better utilise resources. There are challenges with expanding telephone triage services to rural and remote areas, and one that is sometimes not considered are the specific characteristics of the community the service is focussed on [48].

Consistently, the literature reports on nurse expertise and the use of protocols. Most of studies reported on the need for nurse preparation to reach competence; the use of an algorithm or protocol; or reported on the development of an algorithm or tool [20, 35, 37, 40, 43, 52, 53]. Irrespective of the primary focus of the paper, most discussed the need for nurses to have a level of skill and experience to critically assess the patient’s health status and effectively implement the algorithm in practice [35]. That is, the use of protocols, checklists and algorithms rely on a high level of clinical knowledge and experience of the registered nurses [52]. In contrast to the dearth of evidence on mobile phone nurse-led care, the evidence for the employment of experienced registered nurses for health service call lines is clear.

#### Theme 2: Consumer profile

*Demographic and geographic profile of consumers*. The provision of telephone-based support is discussed throughout the literature in the context of people who have limited access to quality healthcare for social, financial, cultural or geographical reasons. Knight et al. found that people who live in rural and remote areas tend to contact their usual health provider directly, rather than utilise formal telephone triage systems. Further, these authors highlight the importance of the telephone as a connection to community, and that ending the phone call did not necessarily reflect the end of the interaction. In many cases, the phone call was the beginning of the episode of care [51]. Interestingly, Cariello [56] reports that users of a phone service rated the level of the nurses’ responsiveness and empathy as more important than reliability and found that education level did not impact on the reported level of satisfaction. This is pertinent in that it is often assumed that people who reside in rural and remote areas have lower levels of formal education [62].

Research on consumers of telephone based medical and health support services focus on population characteristics [19, 27, 39] and the effectiveness of the service for this group; most often defined in terms of compliance [22, 24, 25, 27, 29, 37] or satisfaction [19, 24, 32, 45, 46, 55, 56]. Most of the papers reviewed considered nurse-led support services within the context of a medical model of care, rather than as an autonomous service providing health care/advice within a wellness model [23, 43, 52]. The findings on use, compliance and satisfaction are mixed. For example, Bunn et al., [23] and McKenzie et al., [39] report that marginal populations such as the elderly, the poor or Culturally and Linguistically Diverse (CALD) groups tend to use the services at a lower rate than others, with women and people in remote areas, as opposed to rural regions, higher users of the service. However, McKenzie et al. highlight that despite the percentage of Aboriginal and Torres Strait Islander people who live in rural areas, this population does make frequent use of the telephone triage service, more consistent with the statistics from remote areas. Raknes et al., [30] report that, geographically speaking, as distance from major centres increased, the people accessing the service included fewer women or those seeking assistance for less acute illnesses.

Rurality and distance to major centres influences how the service is utilised by the target population. Studies of nurse-led telephone triage services in rural settings suggest that the more remote the person’s location, the less likely they are to use the service [30, 39, 48, 50]. This appears not to be directly related to social determinant such as gender or socio-economic status. Factors negatively impacting on use in these areas included poor mobile network coverage [50], lack of awareness of services and people in rural centres preferring to directly contact their own health provider [48, 51]. Knight et al. found that telephone support was well used in rural communities when the nurses were known to the community and the call was part of ongoing care, rather than an isolated episode of care, particularly for complex care. Additionally, rural nurses provide the service in a commonly recognised resource-poor context, to achieve best possible outcomes for multiple stakeholders [51]. This then leads to questioning whether the telephone triage role is a specific, substantive role, or is it an extension or part of the nurses’ existing role.

*Consumer satisfaction*. Lee et al. [24] identified the effective use of text messages and social media networks for disseminating information in rural areas, although effectiveness was dependent on individual access to such technology and the age of the consumer. Age and generational factors may also influence how consumers engage with telephone triage/support services. Predictably, Jeong et al. [25] noted that engagement with older adults depended on their health and digital literacy.

The type of advice provided can be divided into three categories; health promotion [19, 25, 31, 42, 43]; assistance in accessing appropriate services and the related symptom triage [43, 48]; or reporting, such as reporting back on out-reach work completed or the outbreak of an infectious disease [23, 26, 33, 34, 37, 43]. Frequency of contact varied in that the telephone service may supplement home visits [28, 38, 57] or simply be a one-off call. Predictably, urban based services in developed countries report receiving more calls after-hours, on weekends or public holidays, and during the winter months [40, 41]. This breakdown, from a developing country’s perspective was not reported in any of the papers in this review.

#### Theme 3 –Outcomes

*Economic/workforce outcomes*. Research on the effectiveness of these services tends to evaluate the quality of the advice provided [35, 56], the impact on Emergency Department admissions/or cost reductions [21, 25, 55, 58] or patient satisfaction [19–21, 24, 32, 40, 41, 44–46, 56, 58]. Ironically, there was very little in the literature that specifically discussed the cost benefits of providing an after-hours telephone advice service to consumers or to health services. For example, only one paper reported on the cost to the consumer [37] while another looked at the associated costs of accessing services for follow-up in rural and remote areas [63].

Several papers report on the effectiveness of telephone services from the perspective of reducing the workload of general practitioners [23, 33, 45]. For example, Bunn et al. [23] identified that nurses can decrease the workload of general practitioners and the need for medically supported home visits, with 50% of consultations handled by a phone call alone, without any increase in adverse events.

The cost of implementing mobile phones as part of health service provision is referred to throughout the literature as cost effective. For example, the use of mobile phones in rural and remote areas are a low-cost, flexible tool in providing healthcare in challenging circumstances [47].

Several papers referred to the ease with which such a service could be provided, in terms of infrastructure and resource provision, noting that the skilled workforce required already existed, with after-hours care being an extension of their professional role. However, Ismail et al. [21] found little impact on Emergency Department attendance and related cost reductions. Only one paper outlined the cost savings from reductions in hospital attendances for non-urgent care and the additional savings that come from patient compliance [37]. This is an anomaly given health budgets are a major consideration for governments. The study reported by Lee et al. [24] makes the point that use of mobile phones is low-cost, accessible and commonly available, utilising an existing resource that most community members already have access to and are familiar with.

*Consumer outcomes*. Reports on telephone based medical and health services are divided between programs implemented in developed economies to increase access, reduce unnecessary admissions (including doctor visits) and cut costs [19, 23, 33, 36, 55, 57]. Studies on consumer satisfaction and compliance similarly report mixed results. Devi and colleagues [19] noted high rates of patient compliance with tele-health advice, as did Mbuagbaw et al. [22], while Egbunike et al. [45] and Karari et al. [50] report that the timeliness of the service was paramount both to satisfaction and compliance. Cariello [56] found lower rates of satisfaction among male patients, but education was not a factor in these outcomes, challenging the idea that compliance with the treatment or advice depends on the patient’s health literacy [25]. A small number of studies within the USA context and other fee-for service systems, noted that factors to do with the patient’s socioeconomic status influenced what care plan the nurse recommended highlighting inequity in service provision [25, 37]. Satisfaction also depends on speciality, with John et al. [27] reporting low levels of satisfaction with nurse-led phone advice for paediatric care.

A number of authors support the notion that mobile phones provide a low-cost, flexible tool for health care in rural areas [47] with Jeong et al. [25] extending this to older adults. These authors also noted that health literacy was an important aspect in relation to the effectiveness of telephone support, and that in the cases of chronic conditions, regular contact by nurses should be planned to improve outcomes. John et al. [27] found that younger patients preferred counselling contact through text technology and see it as more convenient and easier to accept than person to person advice. They also valued the privacy and lack of personal contact. In other studies, patients experiencing social and economic challenges were found to use telephone health services more frequently, whilst women used the service more frequently than men [31, 39].

Jeong et al. [25], whose research related to hypertension and diabetes management in older adults, developed an algorithm that used existing, validated tools to measure patient outcomes. Thus, unsurprisingly, the outcomes variables that this group of researchers measured included, health behaviours, systolic and diastolic blood pressure, fasting blood glucose, hypertension and diabetes self-care behaviours. John et al. [27] evaluated self-care in patients utilising the service, measuring their self-care capacity, psychological adjustment and engagement. These authors found the mobile phone support impacted positively on all these factors.

## Discussion

The evidence found in this review, of the value of nurse-led afterhours mobile phone health care support services for people with chronic conditions, particularly those in rural and remote areas, is inconclusive. The literature retrieved for this review is insufficient to make any judgement on the determinants of a successful nurse-led mobile (cell) phone after- hours service for this population. Part of the difficulty in rating the evidence, is the use of a multiplicity of terms for an array of programs provided to diverse populations for a myriad of health system related reasons. This lack of standardisation is perhaps a reflection of the challenges faced when implementing new services that may not fit within the traditional medical model of care, and that adopt new mobile technologies, and it is possible that the terminology will be refined with time.

It is clear also, that for those patients with chronic and complex conditions, follow up from such calls is important and this could be undertaken within a healthcare team or referral process that allows for on-the-ground follow up, particularly for those people living far from a health service, or those unable to attend a clinic. The follow up could be tailored to the reason for the call, for example it might not require a medical practitioner or nurse or a community trained support worker, particularly where cultural difference between the user and the provider is common. Rural and remote areas would be best served by utilising a call service within existing health service structures, given the fact that community members prefer calling their own health service providers. However, if a call line were to be implemented across a number of services, access to health records or an escalation of service plan, may support better use of clinical expertise. Issues related to this approach would be privacy and access to patient health records, and this would have to be factored into planning.

Another principle emerging from the findings is that rural patients appreciate the relationship developed between them and their health care provider. Studies suggest that they tend to go direct to the health professional they know, are more likely to do so for conditions that are on-going, rather than for one-off health events, and mobile phone services are best accompanied by home visits, as call-centres are seen as impersonal. The transferability of services to meet the distinct needs of people who live in rural and remote areas is also an important consideration. As stated by Knight et al. [51], there is a clear link between telephone-based health services and consumers in rural and remote areas who have an identified need for unscheduled healthcare. However, there should be no one size fits all approach. That is, despite the commonality of geography, there are individual considerations for communities removed from metropolitan areas. Depending on the community identity, health needs will vary accordingly. This is a point not lost on Knight et al. [51], who note that health services in rural and remote areas are inconsistent in “formality and structure around phone-based healthcare provision” (p. 130). Given that there are great differences in distances and level of services available in regions, the phone-based services need to be adapted across multiple interfaces, and in many cases the type of service provided is poorly understood by the consumers, their community and the health professionals who live and work in the area.

Issues related to the health and digital literacy of the user are also highlighted in the existing literature. The literature reports that digital technology is useful for the younger generation but not necessarily for the older populations whose frame of reference is still focused on physically seeing a doctor in the hospital or on medical premises [25, 29, 39]. Despite this, access to health care services has always been difficult or limited for Australian rural populations and mobile telephone services may be an effective alternative to traditional-type health clinics [27, 34]. This is particularly so, given that up until the 1970s remote and rural populations had to rely on the two-way radio and the flying doctor service to communicate with remote doctors/nurses, or had to wait for the remote area nurse to make their monthly visit [64]. Women on remote stations did lean to use the pedal radio suggesting that fear of technology can be overcome. The supposed reluctance to use mobile technology may well be attributable to the reduced access to the technology rather than the reluctance to use it, and the lack of knowledge of such health services [30, 48]. This points to the need to consider making people aware of such services as part of the implementation of any new initiative.

Of further note is the constant reference in the literature of ‘patient compliance’. This term is referred to extensively in different contexts and judged by different measures such as medication and/or treatment compliance. This aspect of telephone triage and outcomes needs further investigation, particularly in relation to patient health outcomes and for organisations, from cost of follow-up or hospital attendances. Patient compliance can relate to many things, but the main reason often cited for non-compliance is the patient and/or their support network do not fully understand the information given to them [29, 37]. This supports the understanding that communication is key to the success of a telephone triage service. It is ironic that in discussions about a service built on technology that success may well depend on the fundamental communication skills of the professional providing the service for the individual.

In summary, the questions posed for this search have been addressed as follows;

- What models of care are used to provide after-hours services to chronic and complex patients?

There is no ‘one-size-fits-all’ answer. The approach to providing after-hours care is dependent on the location, the population and the service availability. The success of managing follow-up care for patients in remote areas is variable, dependent on the patient’s knowledge of modern communication technology and remoteness. Protocols for nurse delivered care have been developed to support a process of care, however evidence supporting chronic and complex conditions is not conclusive.

- Are there models of after-hours care that are led or coordinated by nurses?

Yes. Findings suggest that experienced and/or trained nurses are essential, because of the dislocation of service, i.e. many of the after-hours services are conducted over the telephone, so that it is necessary for nurses to understand what they are hearing and what questions to answer in the case of a change in condition.

- Are there any models or systems of after-hours care for patients with chronic and complex conditions that address geographical isolation?

There are varying models put forward in the literature, none focusing on a disparate group of patients involving complex issues. Findings suggest that models of care need to be developed according to the location and population group it serves.

### Limitations/Further research

Surprisingly no paper discussed the problem of access to digital or telephone technology; the assumption given is that this is not a problem. However, within the Australian context there are still metropolitan, rural and remote areas that cannot reliably access telecommunication.

## Conclusion

This search sought answers to managing chronic and complex conditions across a diverse population group and geographical locations using nurse-led mobile telephone services. Whilst the search itself was rigorous, no definitive answer to the questions were found, except to suggest that models of care for after-hours telephone services needs to be uniquely developed for the population group it serves, inclusive of the geography of the region within which it is located, and the level of media/telecommunication available and understood. However, it is apparent that the provision of an after-hours telephone service, whatever model is used, should be considered within a framework of an evidence-based Chronic Care Model, with elements of each component well suited to the approach. In this way, after-hours telephone services provided by experienced nurses, supported by ongoing professional development and relevant protocols, form part of the ongoing improvement for chronic care management as a health priority.
